# Griscelli Syndrome Type 2 Sine Albinism: Unraveling Differential RAB27A Effector Engagement

**DOI:** 10.3389/fimmu.2020.612977

**Published:** 2020-12-10

**Authors:** Yuta Ohishi, Sandra Ammann, Vahid Ziaee, Katharina Strege, Miriam Groß, Carla Vazquez Amos, Mohammad Shahrooei, Parisa Ashournia, Anahita Razaghian, Gillian M. Griffiths, Stephan Ehl, Mitsunori Fukuda, Nima Parvaneh

**Affiliations:** ^1^ Laboratory of Membrane Trafficking Mechanisms, Department of Integrative Life Sciences, Graduate School of Life Sciences, Tohoku University, Sendai, Japan; ^2^ Institute for Immunodeficiency, Center for Chronic Immunodeficiency, Faculty of Medicine, Medical Center-University of Freiburg, University of Freiburg, Freiburg, Germany; ^3^ Cambridge Institute for Medical Research, University of Cambridge, Cambridge, United Kingdom; ^4^ Department of Pediatrics, Tehran University of Medical Sciences (TUMS), Tehran, Iran; ^5^ Pediatric Rheumatology Research Group, Rheumatology Research Center, Tehran University of Medical Sciences (TUMS), Tehran, Iran; ^6^ Laboratory of Clinical Bacteriology and Mycology, Department of Microbiology and Immunology, KU Leuven, Leuven, Belgium; ^7^ Division of Allergy and Clinical Immunology, Department of Pediatrics, Tehran University of Medical Sciences (TUMS), Tehran, Iran; ^8^ Research Center for Immunodeficiencies, Tehran University of Medical Sciences (TUMS), Tehran, Iran

**Keywords:** Griscelli syndrome type 2 sine albinism, whole-exome sequencing, hemophagocytic lymphohistiocytosis, RAB27A, MLPH/SLAC2-A, MUNC13-4, inborn error of immunity

## Abstract

Griscelli syndrome type 2 (GS-2) is an inborn error of immunity characterized by partial albinism and episodes of hemophagocytic lymphohistiocytosis (HLH). It is caused by *RAB27A* mutations that encode RAB27A, a member of the Rab GTPase family. RAB27A is expressed in many tissues and regulates vesicular transport and organelle dynamics. Occasionally, GS-2 patients with *RAB27A* mutation display normal pigmentation. The study of such variants provides the opportunity to map distinct binding sites for tissue-specific effectors on RAB27A. Here we present a new case of GS-2 without albinism (GS-2 sine albinism) caused by a novel missense mutation (Val143Ala) in the RAB27A and characterize its functional cellular consequences. Using pertinent animal cell lines, the Val143Ala mutation impairs both the RAB27A–SLP2-A interaction and RAB27A–MUNC13-4 interaction, but it does not affect the RAB27A–melanophilin (MLPH)/SLAC2-A interaction that is crucial for skin and hair pigmentation. We conclude that disruption of the RAB27A–MUNC13-4 interaction in cytotoxic lymphocytes leads to the HLH predisposition of the GS-2 patient with the Val143Ala mutation. Finally, we include a review of GS-2 sine albinism cases reported in the literature, summarizing their genetic and clinical characteristics.

## Introduction

Griscelli syndrome type 2 (GS-2; MIM#607624) is an inborn error of immunity (IEI) characterized by partial albinism and the occurrence of acute phases of hemophagocytic lymphohistiocytosis (HLH) (1, 2). *RAB27A* (MIM *603868) gene mutations are responsible for GS-2. RAB27A is a member of the small GTPase family, which is involved in organelle dynamics and intracellular vesicular transport (3, 4). Activated RAB proteins can bind specific effectors and associate with cellular membranes.

RAB27A is highly expressed in melanocytes and a variety of secretory cells, including lymphocytes (5). In mature mouse melanocytes, RAB27A effector/melanophilin (MLPH, also known as SLAC2-A) forms a bridge between RAB27A on mature melanosomes and an actin-based motor myosin Va (MYO5A) and promotes subsequent actin-based melanosome transport (6, 7). Mutations in the *MYO5A* (MIM*160777) encoding MYO5A and *MLPH* (MIM*606526) encoding MLPH/SLAC2-A cause other types of Griscelli syndrome GS-1 (MIM#214450) and GS-3 (MIM#609227), respectively (8, 9). Unlike GS-2 patients, however, GS-1 patients reveal primary neurological problems, and GS-3 patients only exhibit pigmentary dilution.

In cytotoxic T lymphocytes (CTLs), RAB27A controls secretion of cytolytic granules by binding the priming factor MUNC13-4 (2, 10, 11). Mutations in either *RAB27A* or *MUNC13-4* inhibit secretion of granules once they have reached the immunologic synapse. However, little is known about the precise sites of interaction between RAB27A and MUNC13-4 in lymphocytes. Recently, a subpopulation of patients with GS-2 has been reported to present normal pigmentation despite abnormal CTL exocytosis (12–15) (**Table 1**). The study of *RAB27A* mutations that specifically disrupt the interaction of RAB27A with MUNC13-4 but not MLPH provides the opportunity to map distinct binding sites for MUNC13-4 and MLPH on RAB27A. Here we present a new case of GS-2 without albinism caused by a novel missense mutation (Val143Ala) in the RAB27A and characterize its functional cellular consequences. We also show and discuss genetic and clinical findings of other patients with GS-2 sine albinism who reported in the literature.

**Table 1:** Genetic and clinical characteristics of patients with GS-2 sine albinism.

Family	Patient	Age (year)	Sex	Mutation	Protein change	Inheritance	RAB27A interaction with MUNC13-4	RAB27A interaction with MLPH	NK cytotoxicity	NK degranulation	Clinical	Treatment	Outcome	Ref
I	1	2.7	M	c.428T>C	Val143Ala	Homo	Disrupted	Nl	Defective	Defective	CNS HLH	HLH-2004 ATG	Died	Current study
II	2	0.5	M	c.422-424delGAG	Arg141—Val142delinsIle	Homo	Disrupted	Nl	ND	ND	HLH	–	Died	J Allergy Clin Immunol. 2015 May;135(5):1310-8
	3	0.5 m	M	c.422-424delGAG	Arg141—Val142delinsIle	Homo	Disrupted	Nl	Defective	ND	HLH	HSCT	Died	J Allergy Clin Immunol. 2015 May;135(5):1310-8
	4	10.7	F	c.422-424delGAG	Arg141—Val142delinsIle	Homo	Disrupted	Nl	Defective	Defective	HLH	HSCT	Alive	J Allergy Clin Immunol. 2015 May;135(5):1310-8
III	5	7.3	M	c.422-424delGAG c.487A>C	Arg141—Val142delinsIle Ser163Arg	Compound hetero	Disrupted Disrupted	Nl Disrupted	Defective	Defective	HLH	HSCT	Alive	J Allergy Clin Immunol. 2015 May;135(5):1310-8
IV	6	4	F	c.227C>T c.476A>G	Ala76Val Tyr159Cys	Compound Hetero	Disrupted Disrupted	Nl Nl	Defective	Defective	HLH	HSCT	Alive	J Allergy Clin Immunol. 2015 May;135(5):1310-8
V	7	5	M	c.422-424delGAG c.514-518delCAAGC	Arg141—Val142delinsIle Gln172Asnfs	Compound Hetero	Disrupted ND	Nl ND	Defective	Defective	HLH	HSCT	Alive	J Allergy Clin Immunol. 2015 May;135(5):1310-8
VI	8	9	F	c.244C>T	Arg82Cys	Homo	Disrupted	Nl	Defective	Defective	CNS HLH LIP	DEX ATG MMF Alemtuzumab	Died	J Allergy Clin Immunol. 2016 Aug;138(2):599-601
	9	8	M	c.244C>T	Arg82Cys	Homo	Disrupted	Nl	Defective	Defective	No	No	Alive	J Allergy Clin Immunol. 2016 Aug;138(2):599-601
	10	5	M	c.244C>T	Arg82Cys	Homo	Disrupted	Nl	Defective	Defective	No	No	Alive	J Allergy Clin Immunol. 2016 Aug;138(2):599-601
VII	11	14	F	Large 5’ UTR dup/inv/del c.559C>T	SV Arg187Trp	Compound Hetero	ND	ND	Defective	Defective	Neuroinflammation HLH	HLH-2004	Died	J Allergy Clin Immunol. 2018 Jul;142(1):317-321
VIII	12	14.5	M	Large 5’ UTR dup/inv c.239G>C	SV Arg80Thr	Compound Hetero	ND	ND	ND	ND	Neuroinflammation Skin granuloma Lung infiltrate	HLH-2004, ATG	Died	J Allergy Clin Immunol. 2018 Jul;142(1):317-321
IX	13	9	M	Large 5’ UTR dup/inv	SV	Homo	ND	ND	ND	Defective	Neuroinflammation HLH Skin granuloma Lung infiltrate	HLH-2004 HSCT	Alive	J Allergy Clin Immunol. 2018 Jul;142(1):317-321
X	14	8	F	Large 5’ UTR dup/inv	SV	Homo	ND	ND	Defective	Defective	Neuroinflammation Skin granuloma	Steroid MFM HSCT	Alive	J Allergy Clin Immunol. 2018 Jul;142(1):317-321
XI	15	13	M	Large 5’ UTR dup/inv c.550C>T	Arg184*	Compound Hetero	ND	ND	Defective	Defective	Hodgkin lymphoma	GPOH-HD 2002	Alive	J Allergy Clin Immunol. 2018 Jul;142(1):317-321
XII	16	14	M	c.400-401delAA c.74T > G	Lys134Glufs*2 Val25Gly	Compound Hetero	ND	ND	Defective	Defective	Large B cell Lymphoma CNS HLH	HLH-94 HSCT 2 times	Died	Pediatr Blood Cancer. 2020 Aug;67(8):e28312.

ATG, anti-thymocyte globulin; DEX, dexamethasone; CNS, central nervous system; GPOH-HD, German Society of Pediatric Oncology and Hematology-Hodgkin’s Disease; HLH, hemophagocytic lymphohistiocytosis; HSCT, hematopoietic stem cell transplantation; LIP, lymphocytic interstitial pneumonitis; MFM, mycophenolate mofetile; ND, not determined; Nl, normal; SV, structural variant.

## Materials and Methods

### Materials

Horseradish peroxidase (HRP)-conjugated anti-FLAG tag mouse monoclonal (M2) antibody, and anti-FLAG tag antibody-conjugated agarose beads were obtained from Sigma-Aldrich (St. Louis, MO, USA). HRP-conjugated anti-T7 tag mouse monoclonal antibody and anti-T7 tag antibody-conjugated agarose beads were from Novagen™, Merck KGaA (Darmstadt, Germany). Anti-GFP rabbit polyclonal antibody and anti-β-actin mouse monoclonal antibody (C043) were also obtained from MBL (Nagoya, Japan) and Applied Biological Materials (Richmond, BC, Canada), respectively.

### RAB27A Mutation Analysis

Blood and hair samples were obtained with informed consent according to the Institutional Review Boards’ guidelines of the Children’s Medical Center. Genomic DNA was obtained from whole blood by the conventional salting-out method. Whole exome sequencing was performed on a patient sample, as previously described (16). PCR was carried out using primers specific for coding exon 6 of the *RAB27A* gene, as described previously (17). PCR products were directly sequenced using internal primers with an automated ABI PRISM 310 genetic analyzer (PE Applied Biosystems, Norwalk, CT, USA).

### T Cell Culture

Cytotoxic T cell culture (CTL) were prepared by stimulating peripheral blood mononuclear cells (PBMCs) with PHA (1.25 μg/ml) and ∼100 U/ml human IL-2 (produced from a transfected cell line) in the presence of irradiated (30 Gy for 5 min) allogeneic PBMCs as feeder cells. RPMI (Gibco, ThermoFisher, UK) with 5% human serum (Sigma-Aldrich, USA) and ∼100 U/ml human IL-2 was used as culture medium. Every 14 to 18 days, T cells were re-stimulated as above.

### Western Blotting of RAB27A From Patient Cells

T cell cultures were washed in ice-cold sterile PBS and lysed at 2 × 10^7^/ml in lysis buffer [50 mM Tris–HCl (pH7.4), 150 mM NaCl, 1% Triton X-100, 1% NP-40, 2 mM EDTA, and 1x Halt™ protease inhibitors (Thermo Fisher)] for 30 min on ice before cell debris was pelleted by centrifugation at 13,000 for 25 min at 4°C. 22.5 µl cell lysate was mixed with 7.5µl 4x NuPAGE™ LDS reducing sample buffer [Tris base (141 mM), Tris HCl (106 mM), LDS (2%), EDTA (0.51 mM), and 50mM DTT] and heated at 95°C for 5 min before separation on a 4–12% NuPAGE™ Bis-Tris gel in MES running buffer [MES (50 mM), Tris base (50 mM), sodium dodecyl sulfate (SDS) (0.1%), EDTA (1 mM), pH 7.3 (all Thermo Fisher)]. Precision Plus Protein Kaleidoscope Prestained Protein Standards (Bio-Rad, Hercules, CA, USA) were also run. Proteins were transferred to nitrocellulose membranes [Trans-Blot^®^ Turbo™ Mini Nitrocellulose Transfer Packs (Bio-Rad) using the mixed molecular weight program of a semi-dry Trans-Blot Turbo Transfer System (Bio-Rad)]. Membranes were blocked in TBS, 5% non-fat dried milk, and 0.05% Tween-20 (Sigma-Aldrich). Membranes were incubated with primary antibodies (rabbit anti-RAB27A (see ref.12) at 1:1,000 in blocking buffer at 4°C overnight or 1:1,000 rabbit anti-calnexin (C4731, Sigma-Aldrich) for 1 h at room temperature. Membranes were washed 4× for 5 min in TBS, 0.05% Tween, and incubated with 1:10,000 goat anti-rabbit (H+L) HRP labeled secondary antibodies (Thermo Fisher) in blocking buffer for 45 min at room temperature. Membranes were washed as before, developed using ECL Prime Western Blotting solution (Amersham), and imaged using a Bio-Rad ChemiDoc.

### Degranulation Assay

Degranulation of cultured T cells was analyzed at day10 after re-stimulation, and T cells were starved overnight without IL-2 (18). T cells were stimulated with L1210 cells alone or together with 1 µg/ml anti-CD3e (UCHT1, BD Biosciences, UK). Anti-CD107a phycoerythrin, clone H4A3 (eBioscience, UK) was added for the 3-h stimulation time and was again used in combination with anti-CD8 allophycocyanin, clone: MEM-31 (Abcam, UK) for surface staining. Cells were analyzed with flow cytometry (Attune NxT, Thermo Fisher, UK) and FlowJo software.

### Cytotoxic T Lymphocytes Killing Assay

This was as described in (19). In brief, cultured CTLs were added to the P815-NucLight Red-expressing target cells in the presence or absence of 1 μg/ml anti-CD3 antibody (clone UCHT1, catalogue 555330, BD Biosciences—Pharmingen), at a CTL-to-target ratio of 10:1. The killing was measured by the reduction of red fluorescence intensity, indicating target cell death. The assay was measured every 30 min for 4 h using the IncuCyte S3 Live-Cell Analysis System (Essen Bioscience).

### Immunofluorescence Using Artificial Immunological Synapse

Multiwell microscopy slides were cleaned with 70% ethanol for 15 min at room temperature and were coated with 0.01% poly-L-lysine (Millipore Sigma, UK) for 15 min, washed with PBS, and coated with 10 μg/ml hamster humanized anti-CD3 antibody (ChAgly, a gift from Herman Waldmann). Cultured T cells were washed and added in FCS-free IMDM and were allowed to adhere for 12 min. Cells were fixed for 15 min in 4% paraformaldehyde (PFA) (15710-S, Electron Microscopy Systems, USA), permeabilized in 0.1% Triton X-100, and blocked in 2% BSA in PBS (40 min). Samples were labeled for 1 h with primary antibodies [pericentrin (Abcam, UK)] and LAMP1 (H4A3, hybridoma supernatant) and phalloidin followed by fluorophore-conjugated secondary antibodies (donkey anti-mouse 488 and goat anti-rabbit 647, Invitrogen, UK) (1 h at RT) together with phalloidin 568 (Invitrogen, UK). Nuclei were stained for 5 min at RT with Hoechst 33342 (H3570, Thermo Fisher, UK), and samples were mounted in ProLong Diamond Antifade Reagent (P36961, Thermo Fisher, UK). Images were taken with an IX81 Olympus microscope equipped with an Andor Revolution system fitted with a CSU-X1 spinning-disk unit (Yokogawa, UK).

### Plasmid Construction and Site-Directed Mutagenesis

Mutant human RAB27A expression plasmids carrying a Val-to-Ala mutation at amino acid position 143 were produced by inverse PCR techniques essentially as described previously (20) using pEGFP-C1-Rab27A as a template (21) and the following oligonucleotides with a substituted nucleotide (in bold): 5’-GGAGGACCAGAGAGTAG**C**GAAAGAGGAGGAAGCCA-3’ (sense) and 5’-GCTTCCTCCTCTTTC**G**CTACTCTCTGGTCCTCC-3’ (antisense). The *RAB27A* cDNA inserts were excised from the vector with appropriate restriction enzymes and subcloned into the pEF-FLAG tag mammalian expression vector (22) and pMRX-IRES-EGFP tag retrovirus vector (23) as described previously. Other expression plasmids, including pEF-T7-MUNC13-4, pEF-T7-SLP2-A, pEF-T7-MLPH/SLAC2-A, were prepared as described previously (24, 25).

### Cell Cultures, Transfections, and Stable Expression of RAB27A

The black mouse-derived immortal melanocyte cell line melan-a and *ashen* mouse-derived immortal melanocyte cell line melan-ash were obtained from the Wellcome Trust Functional Genomics Cell Bank and cultured as described previously (26–28). COS-7 cells and Plat-E cells [a kind gift from Toshio Kitamura (The University of Tokyo, Tokyo, Japan)] were maintained at 37°C under 5% CO_2_ in DMEM (FUJIFILM Wako Pure Chemical, Osaka, Japan) containing 10% fetal bovine serum and antibiotics. Retrovirus production and infection were performed, as described previously (29). Stable melan-ash cell lines were obtained by blasticidin selection (10 µg/ml for 5–7 days).

### Immunofluorescence Analysis and Melanosome Distribution Assay

Two immortal mouse melanocyte cell lines were cultured on coverslips and fixed with 4% PFA for 10 min. The coverslips were incubated for 1 h with DAPI. The samples were mounted using ProLong Diamond Antifade Mountant (Thermo Fisher Scientific, Waltham, MA). Infected cells were identified by EGFP fluorescence, and their fluorescence images, together with the corresponding bright-field images, were captured at random with an FV1000D confocal fluorescence microscope and Fluoview software (Olympus, Tokyo, Japan). Melanosome distribution was assessed by examination of images of infected melan-a/ash cells (more than 25 cells/dish, three independent dishes for each transfection). Cells in which more than 50% of the melanosomes were present around the nucleus were judged to be aggregated as described previously (28).

### Co-Immunoprecipitation Assay in COS-7 Cells

COS-7 cells were co-transfected for 24 h with pEF-FLAG-RAB27A (wild-type or a Val143Ala mutant) and pEF-T7-MUNC13-4, pEF-T7-SLP2-A, or pEF-T7-MLPH/SLAC2-A by using Lipofectamine 2000 (Invitrogen, Thermo Fisher Scientific). The transfected cells were lysed with a lysis buffer [50 mM HEPES-KOH, pH 7.2, 150 mM NaCl, 1 mM MgCl_2_, and 1% Triton X-100 supplemented with complete EDTA-free protease inhibitor mixture (Roche, Basel, Switzerland)]. The cell lysates were incubated for 1 h at 4°C with anti-FLAG or anti-T7 tag antibody-conjugated agarose beads. The beads were washed three times with a washing buffer (50 mM HEPES-KOH, pH7.2, 150 mM NaCl, 1 mM MgCl_2_, and 0.1% Triton X-100), and proteins bound to the beads were analyzed by 10% SDS-polyacrylamide gel electrophoresis (PAGE) followed by immunoblotting with HRP-conjugated anti-FLAG and anti-T7 tag antibodies. Immunoreactive bands were visualized by enhanced chemiluminescence.

### Statistical Analysis

Statistical tests were performed using Tukey’s test, and *p* values <0.05 were considered statistically significant.

## Results

### Clinical Phenotype

**Figure 1:**
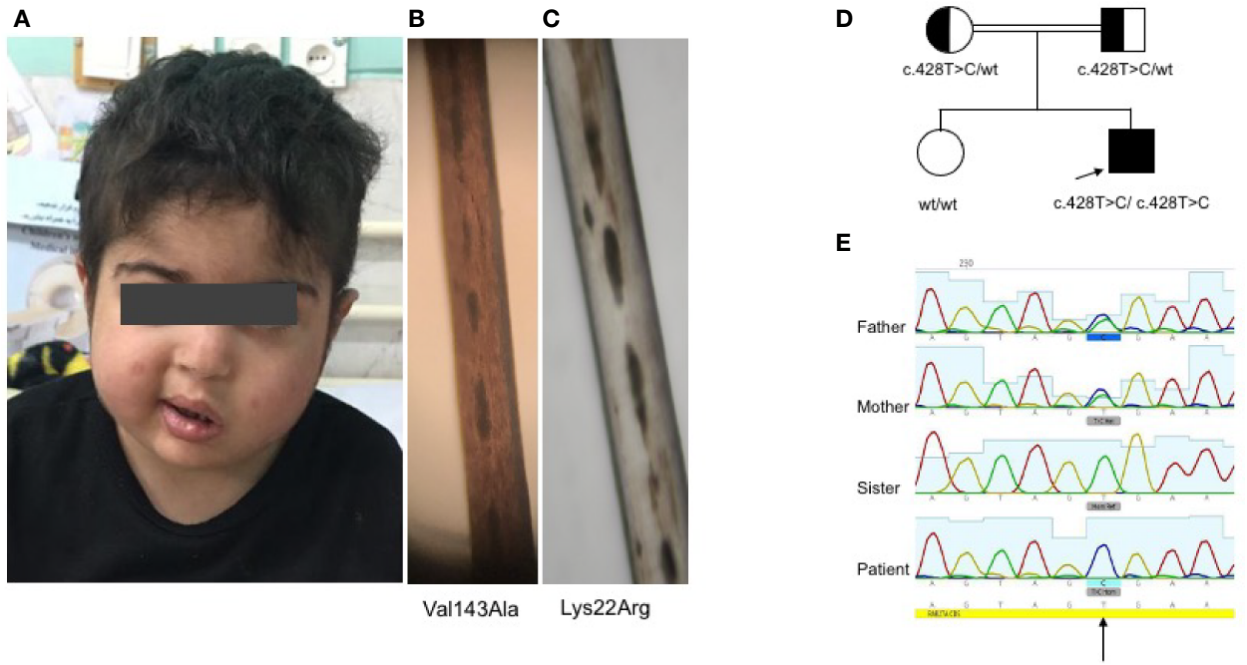
Clinical and genetic findings of the patient **(A)**. Normal complexion with left-sided Bell’s palsy **(B)**. Fairly normal hair pigmentation of the patient (Val143Ala) determined by light microscopy compared to **(C)** abnormal clumps of melanin characteristic of GS-2 (Lys22Arg) **(D, E)**. Pedigree of the family showing the familial segregation of *RAB27A* mutation and chromatograms pertinent to each family member.

The patient was a boy of Iranian origin and was born to consanguineous parents. Informed consent was obtained from the parents. He had no albinism, and his hair shafts showed fairly normal pigmentation (**Figures 1A–C**). Laboratory data revealed a white blood cell count of 360 cells/µL, an absolute neutrophil count of 10 cells/µl, hemoglobin of 7.7 g/dl, and platelets of 23,000 cells/µl. Serum chemistry revealed triglyceride of 267 mg/dl (normal <160), ferritin of 23,840 ng/ml (normal<500), and fibrinogen of 127 mg/dl (normal >150). The bone marrow exam demonstrated mild dyserythropoiesis without any hemophagocytic changes. Epstein-Barr virus and cytomegalovirus load in plasma were negative. The patient fulfilled five HLH criteria, and secondary causes were excluded, so he was started on an HLH-94 protocol and referred to receive an allogenic hematopoietic stem cell transplantation. The disease was fairly well controlled when he presented at 53 months with left-sided Bell’s palsy. Brain MRI indicated facial nerve demyelinating disease. The cerebrospinal fluid (CSF) analysis was unremarkable. Methylprednisolone (30 mg/kg/day for three consecutive days) and a dose of anti-thymocyte globulin (5 mg/kg/day for three days) resulted in resolution of symptoms. Eight months later, he presented with seizure and drowsiness. CSF analysis showed lymphocytic pleocytosis and increased protein levels. Intrathecal methotrexate/hydrocortisone was started. However, the neurologic disease progressed over the next months, and he developed liver failure and severe cytopenias, leading to his death one month later.

### Identification of RAB27A Mutation

Whole exome sequencing revealed a homozygous missense c.428T>C (Val143Ala) variant in exon 6 of *RAB27A* (NM_183236). No important mutations in other genes relevant to HLH were detected, including *UNC13D*, *STX11*, *STXBP2*, *LYST*, *PRF1*, *SH2D1A*, *XIAP*, *ITK*, *CD27*, *AP3B1*, *NLRC4*, and *MAGT1*. Similarly, known IEI-related genes lacked any rare deleterious mutations. Sanger sequencing confirmed the homozygous *RAB27A* mutation in the patient, while a heterozygous mutation was found in parents and homozygous wild type in the healthy sister (**Figures 1D, E**). This is a novel mutation with no allele reported in public databases such as gnomAD. Based on the PolyPhen-2 amino acid change predictor, this mutation is likely deleterious. The combined annotation dependent depletion (CADD) score of this mutation is 25.7; higher than the mutation significance cutoff (MSC) of 4, pertinent to *RAB27A* (using HGMD with 99% confidence interval) (30).

### Degranulation and Immune Synapse Formation Assays

**Figure 2:**
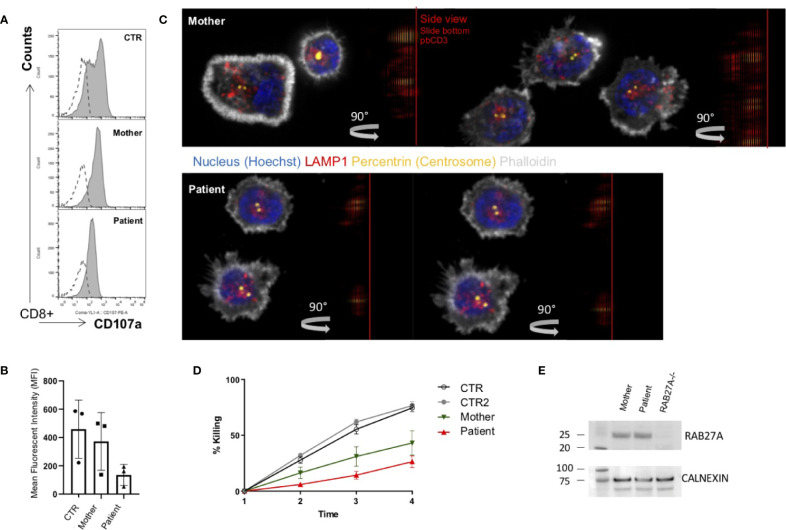
Impaired degranulation and killing of patient cytotoxic T cells **(A)**. *Ex vivo* degranulation of cultured cytotoxic T cells from the patient, heterozygous mother, and a healthy control after incubation with medium (dotted line) or with 1 µg/ml anti-CD3 + L1210 cells was measured by surface expression of CD107a using flow cytometry **(B)**. Collected mean intensity fluorescence (MFI) of 3 independent experiments showing the difference (delta MFI) between unstimulated and stimulated cells **(C)**. Lytic granule mobilization to an artificial immunological synapse was normal of patient and mother CTLs. Microscope slides were coated with 10 µg/ml anti-CD3, CTLs were settled for 10 min, PFA fixed, permeabilized with saponin, and stained for actin (white), pericentrin (yellow), and lytic granules (red). Shown are single slices from the artificial synapse interface formed on the microscope slide and a 90°C rotation of all sliced showing the location of the lytic granules close to the pericentrin. Bottom of the microscope slide in the 90°C side view (plate-bound CD3 localization), is indicated by a red line **(D)**. Percentage lysis of P815-NucLight Red targets over time measured by IncuCyte killing assay using two healthy controls (CTR), the mother and patient CTLs overtime measured for 4 h **(E)**. Protein analysis by western blotting showed normal protein expression of RAB27A in the mother and the patient’s CTLs. To show specificity of the RAB27A antibody a human RAB27A knockout (RAB27-/-) CD8^+^ cell line was run in parallel.

In line with other homozygous mutations in the *RAB27A*, the patient’s CTLs showed impaired degranulation. In contrast, the mother’s CTLs with the heterozygous mutation showed slightly reduced to normal degranulation compared to healthy control (**Figure 2A**). Degranulation was performed in 3 independent experiments (**Figure 2B**). Furthermore, we analyzed the transport to the immunological synapse (IS) of the lytic granules by generating an artificial synapse on microscopy slides using glass bound anti-CD3. As an indication for synapse formation, we stained for actin using phalloidin. At the synapse, actin is cleared, the microtubule-organization center or centrosome (stained by pericentrin) moves to the synapse, and the lytic granules are transported along the microtubules to the immunological synapse. In line with other reports of lytic granule polarization (11, 31), the transport to the immunological synapse was normal (**Figure 2C**). This is highlighted by the 90° side view of the cell, demonstrating the localization of the lytic granules close to the centrosome and the immunological synapse and not far back in the cell body. However, the release of the lytic granules was impaired, and the patient’s CTLs showed the reduced killing of targets (**Figure 2D**). Despite the functional impairment, the mutated protein of RAB27A was expressed stably in the CTLs of the patient (**Figure 2E**), pointing to an impaired binding activity to MUNC13-4.

### Subcellular Localization and Function of RAB27A(Val143Ala) in Cultured Mouse Melanocytes

**Figure 3:**
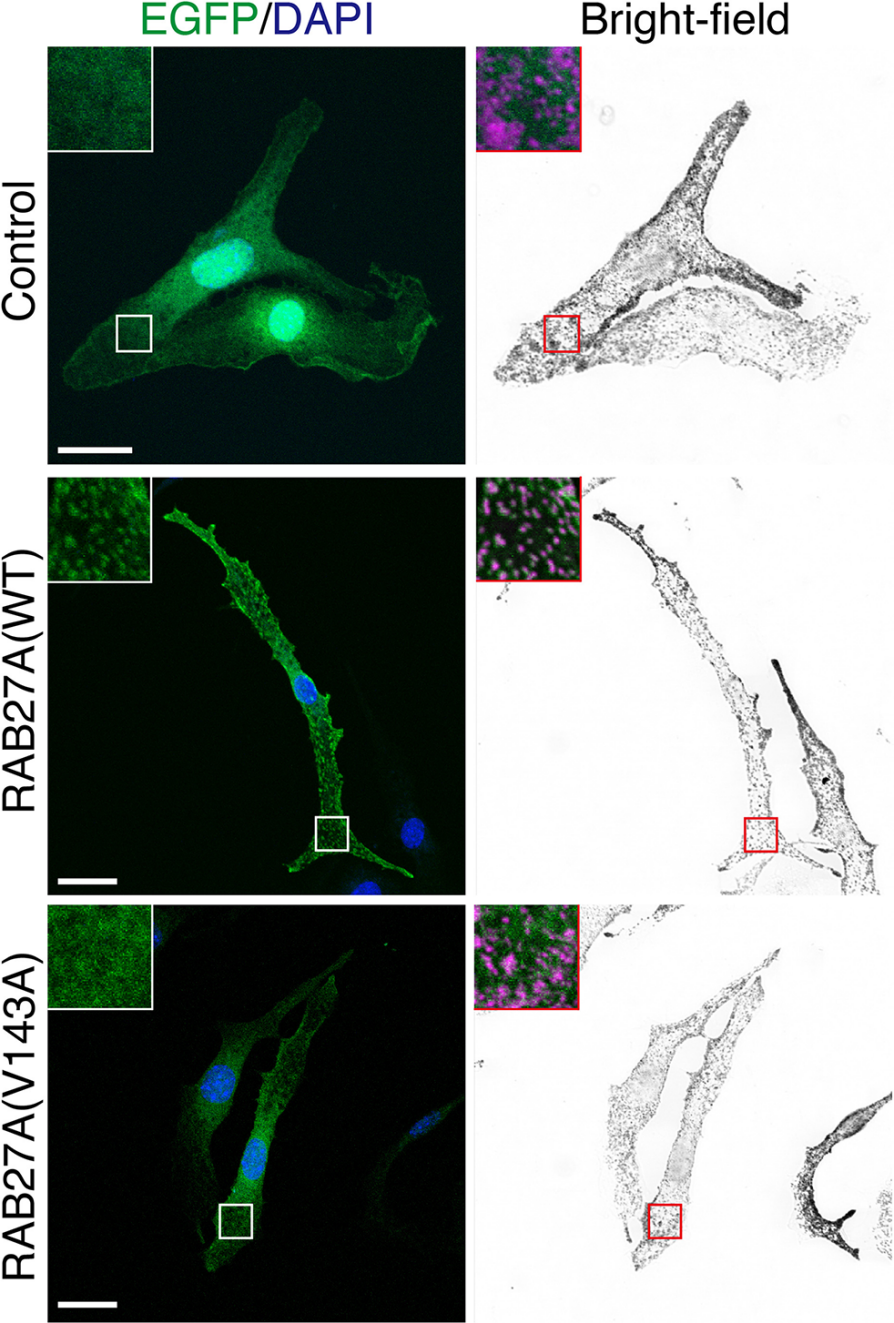
Subcellular localization of RAB27A(Val143Ala) mutant and its effect on melanosome distribution in wild-type melanocytes. Melan-a cells stably expressing either EGFP alone (top panels), EGFP-RAB27A (WT, wild-type) (middle panels), or EGFP-RAB27 (Val143Ala) (indicated as V143A; bottom panels) (EGFP in green and DAPI in blue). The insets show magnified views of the boxed areas (melanosomes are pseudo-colored in magenta). Note that RAB27A (Val143Ala) was mostly present in the cytosol and only weakly co-localized with melanosomes, whereas wild-type RAB27A was present on melanosomes. Scale bars, 20 µm.

To evaluate the effect of the Val143Ala mutation on the melanosomal localization of RAB27A, an EGFP (enhanced green fluorescence protein)-tagged RAB27A (Val143Ala) mutant was stably expressed in black mouse-derived melanocytes [melan-a cells (27)] using retroviral infection (**Figure 3**). While wild-type EGFP-RAB27A showed a clear punctate localization on melanosomes (**Figure 3**, middle panels), EGFP-RAB27A (Val143Ala) was only weakly associated with melanosomes (**Figure 3**, lower panels) and some portions of the mutant protein seemed to localize in the cytosol similar to EGFP alone (**Figure 3**, top panels). As EGFP-RAB27A (Val143Ala) had no effect on the distribution of melanosomes, in contrast to EGFP-tagged mouse RAB27A (Gln78Leu) previously described (32), the Val143Ala mutation did not confer a dominant-negative function on RAB27A in melanosome transport. These results suggested that while the RAB27A (Val143Ala) mutant partially impaired localization, it did not produce a dominant-negative phenotype when over-expressed in melanocytes.

**Figure 4:**
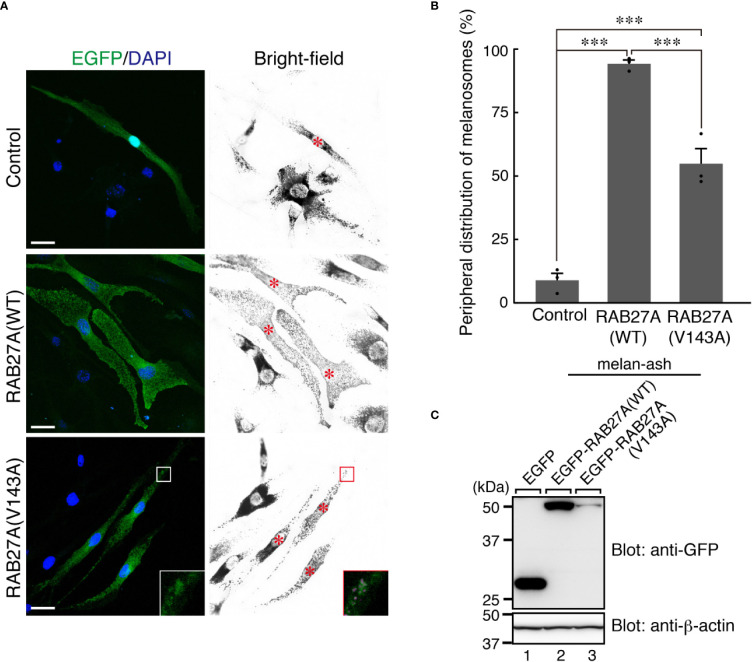
RAB27A (Val143Ala) partially restored peripheral melanosome distribution in RAB27A-deficient melanocytes **(A)**. Melan-ash cells stably expressing either EGFP alone (top panels), EGFP-RAB27A (WT, wild-type) (middle panels), or EGFP-RAB27A (Val143Ala) (indicated as V143A; bottom panels) by retrovirus infection (EGFP in green and DAPI in blue). The insets show magnified views of the boxed areas (melanosomes are pseudo-colored in magenta) and indicate the association of EGFP-RAB27A (Val143Ala) with melanosomes in melan-ash cells. Note that expression of wild-type RAB27A in melan-ash cells completely rescued the perinuclear aggregation phenotype (i.e., peripheral melanosome distribution), whereas expression of RAB27A (Val143Ala) did partially. Scale bars, 20 µm **(B)**. Quantification of the cells with peripheral melanosome distribution shown in **(A)**. The results are expressed as the percentages of infected cells exhibiting peripheral melanosome distribution (means ± S.E. of three determinations; n > 25 in each determination). ***p < 0.001 (Tukey’s test) **(C)**. The protein expression level of EGFP-RAB27A (WT) and EGFP-RAB27A(Val143Ala) in blasticidin-selected melan-ash cells. Cell lysates were subjected to 10% sodium dodecyl sulfate polyacrylamide gel electrophoresis (SDS-PAGE), followed by immunoblotting with the antibodies indicated. The positions of the molecular mass markers (in kilodaltons) are shown on the left.

Partial melanosomal localization of EGFP-RAB27A (Val143Ala) in melan-a cells further prompted us to investigate whether this mutant supports melanosome transport in the absence of an endogenous mouse RAB27A. To this end, we stably expressed the Val143Ala variant in RAB27A-deficient immortalized melanocytes [melan-ash cells (26);] that typically showed perinuclear aggregation of melanosomes (**Figure 4A**, upper right panel). When the wild-type EGFP-RAB27A was stably expressed in the melan-ash cells, it was clearly localized to melanosomes (**Figure 4A**, middle panels), and more than 90% of melan-ash cells expressing EGFP-RAB27A restored the peripheral distribution of melanosomes (**Figure 4B**). On the other hand, unlike in the case of melan-a cells, EGFP-RAB27A (Val143Ala) showed punctate localization on melanosomes in melan-ash cells (**Figure 4A**, lower panels) and rescued the perinuclear aggregation phenotype, although less efficiently than EGFP-RAB27A (**Figure 4B**). Such partial rescue effect of EGFP-RAB27A (Val143Ala) is likely to be attributable to its lower expression level in melan-ash cells (**Figure 4C**) because there is a clear positive correlation between the protein expression level of RAB27A (Val143Ala) and rescue efficiency (data not shown). However, we also found another phenotype even in the rescued cells: melanosomes did not accumulate in the peripheral area of RAB27A (Val143Ala)-expressing melan-ash cells when compared to the wild-type RAB27A-expressing cells (**Figure 4A**).

### Effect of the Val143Ala Mutation of RAB27A on Binding Activity Toward SLP2-A, MLPH/SlLAC2-A, and MUNC13-4

**Figure 5:**
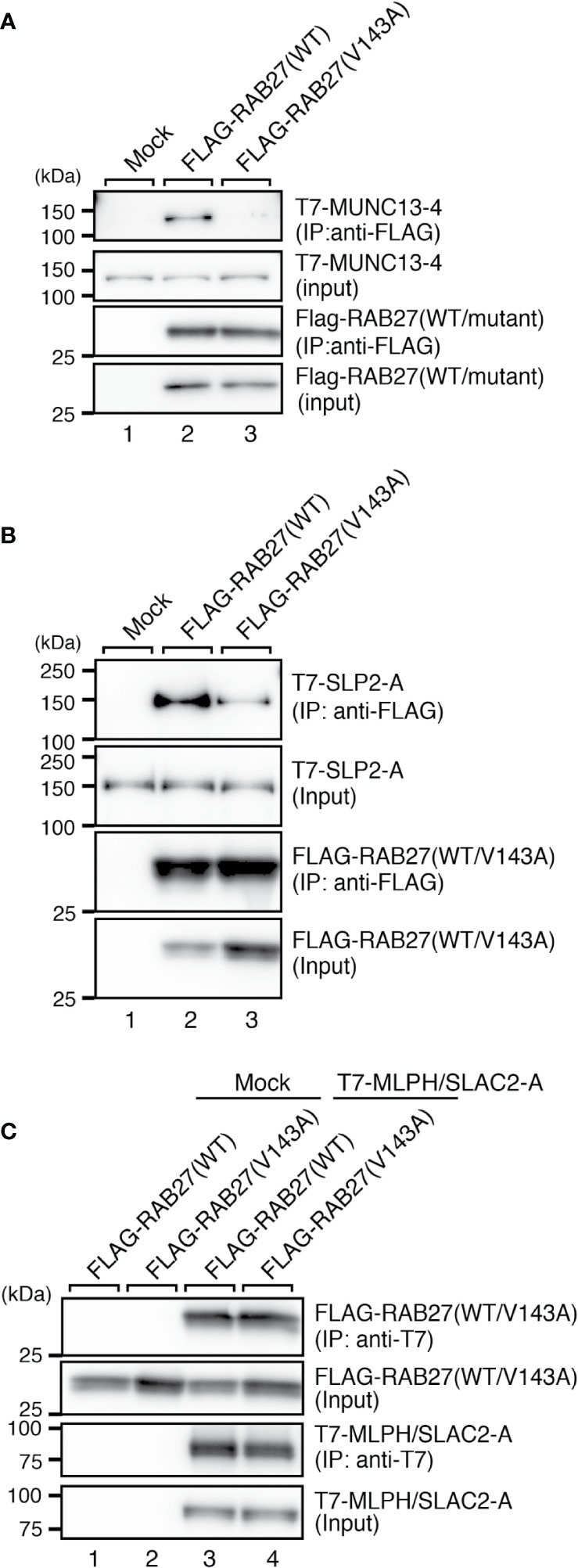
RAB27A effector binding activities of RAB27A(Val143Ala). Interaction between T7-MUNC13-4 and FLAG-RAB27A (WT or Val143Ala; indicated as V143A) **(A)**, T7-SLP2-A and FLAG-RAB27A (WT or Val143A) **(B)**, or T7-SLAC2-A and FLAG-RAB27A (WT or Val143Ala) **(C)**, in COS-7 cell lysates was analyzed by co-immunoprecipitation assays using anti-FLAG tag antibody-conjugated agarose beads **(A, B)** or anti-T7 tag antibody-conjugated agarose beads **(C)**. Co-immunoprecipitated T7-MUNC13-4 and T7-SLP2-A (or FLAG-RAB27A) and immunoprecipitated FLAG-RAB27A (or T7-MLPH/SLAC2-A) were detected by immunoblotting with HRP-conjugated anti-T7 tag antibody and anti-FLAG tag antibody (or anti-FLAG tag antibody and anti-T7 tag antibody), respectively. The positions of the molecular mass markers (in kilodaltons) are shown on the left. Note that the Val143Ala mutation of RAB27A dramatically reduced the binding activity toward MUNC13-4 and SLP2-A.

Because RAB27A is known to regulate actin-based melanosome transport in melanocytes and lytic granule exocytosis in cytotoxic T lymphocytes through interaction with cell-type-specific or tissue-specific effectors (33), we finally investigated the effect of the Val143Ala mutation of RAB27A on binding activity toward its effectors. We selected three well-known RAB27A effectors, i.e., mouse SLP2-A, MLPH/SLAC2-A, and MUNC13-4, that function in melanocytes and/or cytotoxic T lymphocytes (6, 7, 34–38) and performed co-immunoprecipitation assays in COS-7 cells by co-expressing FLAG-tagged RAB27A and T7-tagged RAB27A effectors. The results showed that the Val143Ala mutation dramatically reduced the binding activity toward MUNC13-4 and SLP2-A (**Figures 5A, B**). Intriguingly, however, it had no effect on the binding activity toward MLPH/SLAC2-A (**Figure 5C**). Consistent with the normal MLPH/SLAC2-A binding activity of RAB27A (Val143Ala), its expression in melan-ash cells mostly rescued the perinuclear aggregation phenotype, i.e., the typical MLPH/SLAC2-A-deficient phenotype (**Figures 4A, B**). However, because RAB27A(Val143Ala) showed decreased SLP2-A binding activity (**Figure 5B**), melanosomes did not accumulate in the peripheral area in RAB27A (Val143Ala)-expressing melan-ash cells (**Figure 4A**, bottom right panel, boxed), which is a typical peripheral dilution phenotype observed in SLP2-A-deficient melanocytes (38). Taken together, these results indicated that the Val143Ala mutation of RAB27A differently affected binding activity toward RAB27A effectors.

## Discussion

In the present study, we biochemically and functionally analyzed a novel homozygous *RAB27A* mutation (Val143Ala) found in a GS-2 patient without albinism. Defective CTL degranulation and cytotoxic functions confirm the functional relevance of the mutation for lymphocyte cytotoxicity, explaining the HLH predisposition phenotype of the patient.

The effect of the Val143Ala mutation in melanocytes was clearly different from that of two previously characterized GS-2 mutations (Lys22Arg and Ile44Thr) with partial albinism in our previous studies. The Lys22Arg mutation caused cytosolic localization of EGFP-RAB27A (Lys22Arg) because of its defect in GTP binding activity, whereas the Ile44Thr mutation had no effect on the melanosomal localization of RAB27A (Ile44Thr) (25). The Val143Ala mutation of RAB27A may also affect the folding of RAB27A, which consequently destabilizes the mutant protein leading to its degradation or its inefficient melanosome targeting, at least in melanocytes. However, since the decreased expression of RAB27A protein was not observed in the patient’s CTLs, certain chaperones that express only in CTLs might stabilize mutant RAB27A protein. In contrast to the Val143Ala mutation, either the Lys22Arg or Ileu44Thr mutation completely impaired MLPH/SLAC2-A-binding activity (25), which is presumably the primary cause of partial albinism of these GS-2 patients.

We noted the fact that Val143 of human RAB27A is located at a bend region between the β5 strand and the α4 helix of the common RAB GTPase structure and that it is not located in the consensus phosphate/magnesium-binding motifs or guanine base-binding motifs. However, Val in this position is invariant among the RAB27 subfamily proteins from different species (25) and highly conserved among the mammalian RAB family proteins. Moreover, Val143 of RAB27A/B does not directly interact with either MLPH/SLAC2-A or SLP2-A based on the crystal structure of the MLPH/SLAC2-A-SHD–RAB27B complex (39) and SLP2-A-SHD–RAB27A complex (40). We thus suggest that the Val143Ala mutation may indirectly affect the spatial position of critical residues located at a neighboring α-helical region (e.g., α5 helix) for SLP2-A binding. Further detailed structural analysis is necessary to determine the Val143Ala mutation’s impact on the RAB27A–SLP2-A interaction.

**Figure 6:**
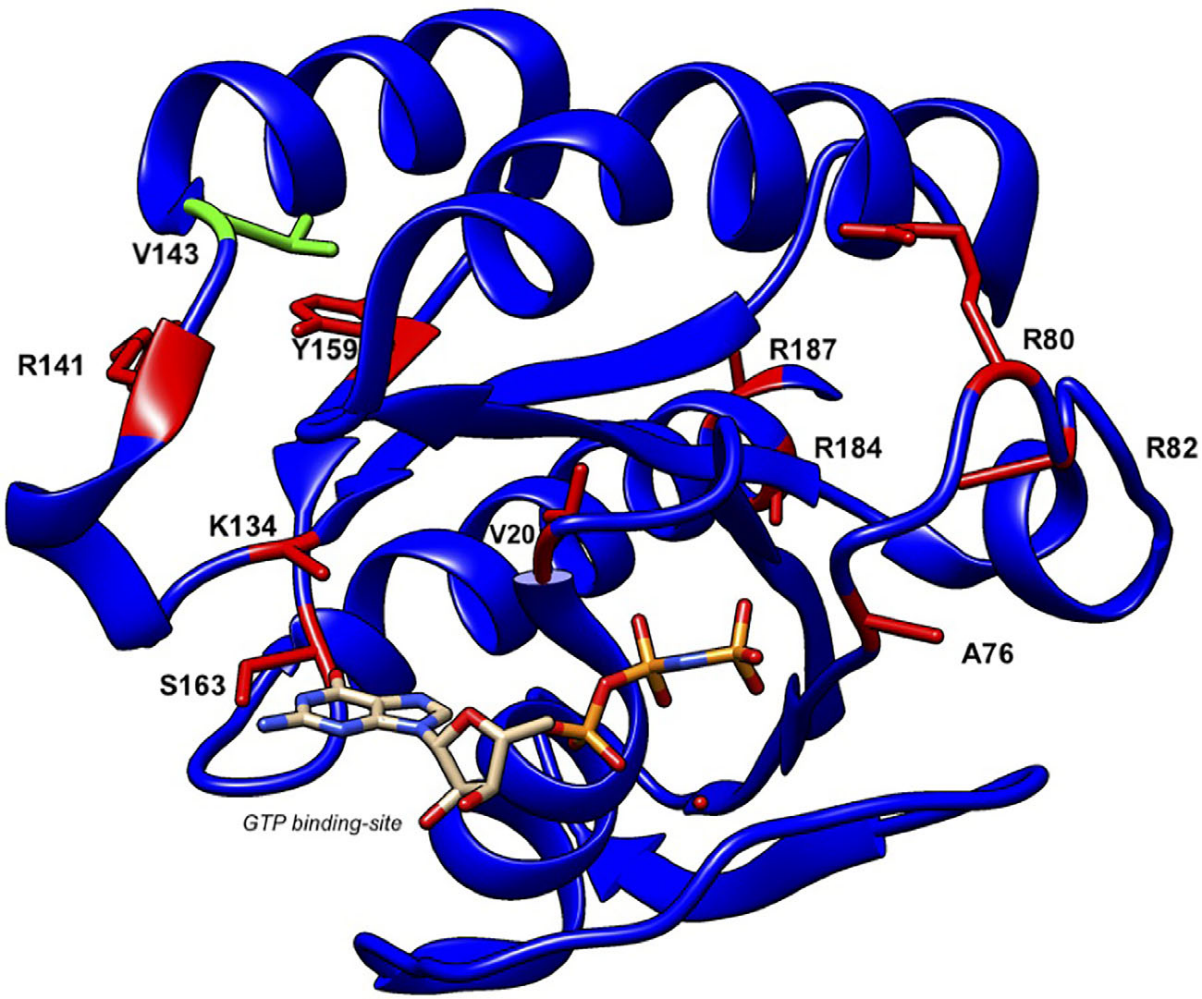
Structure of human RAB27A and localization of mutations in patients with GS-2 sine albinism. A 3D model of human RAB27A complexed with GTP was depicted by UCSF CHIMERA (https://www.cgl.ucsf.edu/chimera/). Val143 and other amino acids that were mutated in patients with GS-2 sine albinism were highlighted in green and red, respectively.

To date, a total of 16 patients from twelve families have been reported to present with GS-2 sine albinism (12–15). The study of these *RAB27A* variants suggests potential mechanisms underlying this atypical GS-2. First, mutations at residues Arg80, Arg82, Arg141, and Val143 that lie on the surface of the protein may directly interact with MUNC13-4 while these and other internal residues may lead to conformational changes in RAB27A that disrupt this binding (12, 13) (**Figure 6**). Other mutations, such as Arg184, seem to disrupt protein stability, leading to a loss of RAB27A in patient cells (14). Additionally, the study of a 5’ untranslated region structural variant linked the lack of hypopigmentation to differential *RAB27A* transcription start sites usage between lymphocytes and melanocytes (14). The study of compound heterozygous *RAB27A* variants showed that the presence of one such allele is sufficient to preserve normal pigmentation (12, 14). Clinically, the age of onset and clinical presentation of HLH seem to be similar to typical GS-2. GS-2 sine albinism patients present with a high rate of neuroinflammation (44%). Lung involvement and skin granuloma were presented in three and two patients, respectively. Interestingly, two patients presented with B cell lymphoma, which is not reported in typical GS-2. The importance of such clinical findings in the context of presented mutations is unclear at present.

In conclusion, the results of our analyses of the Val143Ala mutation of RAB27A can explain the phenotype of the GS-2 patient without albinism. The Val143Ala mutation impairs both the RAB27A–SLP2-A interaction and RAB27A–MUNC13-4 interaction, but it has no effect on the RAB27A–MLPH/SLAC2-A interaction that is crucial for skin and hair pigmentation (8, 41). Because SLP2-A knockout mice showed neither immunodeficiency (34) nor severe hypopigmentation (42), certain compensation for the SLP2-A-mediated docking step would occur in CTL secretion and melanosome transfer to keratinocytes *in vivo*. We thus conclude that disruption of the RAB27A–MUNC13-4 interaction in cytotoxic lymphocytes leads to the impaired cytotoxicity, the immunodeficiency, and the predisposition to HLH of the GS-2 patient with homozygous Val143Ala mutation, as has been shown in *jinx* mice and type 3 familial hemophagocytic lymphohistiocytosis (FHL3) patients (31, 37, 43). *RAB27A* mutations should be investigated in patients with suspected HLH disease, even if there is no pigmentary dilution.

## Data Availability Statement

The datasets presented in this study can be found in online repositories. The names of the repository/repositories and accession number(s) can be found below: https://www.ncbi.nlm.nih.gov/genbank/, MW049352.

## Ethics Statement

The studies involving human participants were reviewed and approved by The IRB of Children’s Medical Center affiliated to TUMS approved this study (IR.TUMS.CHMC.REC.1399.080). Written informed consent to participate in this study was provided by the participants’ legal guardian/next of kin. Written informed consent was obtained from the minor(s)’ legal guardian/next of kin for the publication of any potentially identifiable images or data included in this article.

## Author Contributions

YO performed the research on melanocytes and binding assays in COS-7 cells, analyzed data, and wrote the manuscript. SA performed killing, artificial synapse, and degranulation assays and wrote the manuscript. VZ, PA, and AR followed the patient clinically. KS and MG performed killing and degranulation assays. CA performed patient western blot. MS performed whole-exome sequencing and Sanger sequencing. GG supervised the CTL derivation, killing, and degranulation assays. SE supervised degranulation and artificial synapse assay. MF supervised the melanocyte research and wrote the manuscript. NP followed the patient, proposed the study, and wrote the manuscript. All authors contributed to the article and approved the submitted version.

## Funding

This work was supported in part by Grant-in-Aid for Scientific Research (B) from the Ministry of Education, Culture, Sports, Science and Technology (MEXT) of Japan (grant number 19H03220 to MF), and by Japan Science and Technology Agency (JST) CREST (grant number JPMJCR17H4 to MF). Also, funding supporting SA, KS, and GG from the Wellcome Trust 10390 and 100140. SE and MG were funded by the Deutsche Forschungsgemeinschaft (DFG, German Research Foundation)—SFB1160/2—Project A01. NP is an associate professor of pediatrics at the Tehran University of Medical Sciences (TUMS).

## Conflict of Interest

SA worked as a scientific advisor for Sobi. The IRB of Children’s Medical Center affiliated to TUMS approved this study (IR.TUMS.CHMC.REC.1399.080).

The remaining authors declare that the research was conducted in the absence of any commercial or financial relationships that could be construed as a potential conflict of interest.

The reviewer HK declared a past co-authorship with one of the authors SE to the handling editor.

The reviewer AF declared a past co-authorship with one of the authors SE to the handling editor.
